# Evaluation of cognitive functions in a group of Egyptian recovered COVID-19 patients

**DOI:** 10.1186/s43045-023-00308-2

**Published:** 2023-05-22

**Authors:** Hadir E. E. M. Allam, Abd El-Nasser M. Omar, Maha M. Sayed, Amira N. El-Batrawy, Nesreen M. Mohsen, Abdel Gawad Khalifa, Fiby F. Ghobrial

**Affiliations:** Neuropsychiatry Department, Okasha Institute of Psychiatry, Ain Shams University, Cairo, Egypt

**Keywords:** COVID-19, Cognitive impairment, Recovered COVID patients, WCST, WMS, Anxiety and depression

## Abstract

**Background:**

Numerous investigations have found that cognitive deficits in COVID-19 survivors may be reversible; hence, early detection is essential. These cognitive deficiencies should be targeted with scaled cognitive therapies that can be widely used even in patients’ homes, supporting the best possible cognitive and functional outcomes. In the meanwhile, it has been observed that COVID-19 patients may experience worry, fear, depression, and other mental health problems. Therefore, subjective cognitive difficulties may be due to emotional discomfort. As a result, these data highlight the significance of early diagnosis of anxiety symptoms and depression symptoms in COVID-19 patients in order to prevent subsequent cognitive problems. All patients were selected in accordance with the case definition and used the following tools after 1, 3, and 6 months after being cleared of COVID-19 infection: developed questionnaire for both clinical and demographic data, the Wisconsin Card Sorting Test, the Wechsler Memory Scale-Revised, the Wechsler Adult Intelligence Scale, the Hamilton Anxiety Rating Scale, the Beck Depression Inventory, and Structured Clinical Interview for DSM-IV disorders.

**Results:**

Fifty patients were participated in this study from both gender, different levels of education, and the major group was nonsmokers (82%). A total of 88% of participants had confirmed COVID-19, and 12% had contact with them. Wisconsin Card Sorting Test for preservative parameters revealed that the 2nd follow-up showed nonsignificant comparison to the 1st follow-up, while the 3rd showed highly significant comparison to the 1st follow-up. While for non-preservative errors, the 2nd follow-up showed significant comparison to the 1st, while the 3rd showed highly significant comparison to the 1st follow-up. Conceptual level response parameters showed that both the 2nd and the 3rd follow-ups showed nonsignificant comparison to the 1st follow-up. There was no significant correlation between Hamilton Anxiety Scale (HAS) and any parameter of Wisconsin Card Sorting Test or any parameter of Wechsler Memory Scale-Revised.

**Conclusions:**

While there was negative impact of COVID-19 infection on cognitive functions in Egyptian recovered COVID-19 patients which improves gradually by time, there was nonsignificant correlations between anxiety symptoms, depressive symptoms, and Wisconsin Card Sorting Test as well as Wechsler Memory Scale-Revised parameters in tested individuals through three consecutive follow-ups of COVID-19 in Egypt. Further testing using other scales or larger sample is mandatory to elucidate further potential impact of COVID-19 on cognitive functions of recovered patients.

## Background

The Severe Acute Respiratory Coronavirus 2 (SARS-CoV-2) is a new coronavirus that has been present in Wuhan, China, from December 2019. The World Health Organization (WHO) has designated the SARS-CoV-2 viral outbreak as coronavirus disease 2019 (COVID-19) [1]. Following the discovery that COVID-19 infection is transmitted between humans by particles or direct engagement [2–4], the WHO proclaimed it to be a global public health emergency due to its global outbreaks and severe burden [5]. It has been reported that older age, male sex, African ethnicity, a lower social position, and several preexisting medical illnesses are risk factors for both infections with COVID-19 and its sequelae [6, 7]. Additionally, genetic variations were discovered to be one of the reasons why people respond to the SARS-CoV-2 differently, either directly by affecting virus entrance and replication or indirectly by predisposing them to illnesses that speed up the advancement of COVID-19 [8].

The definition of cognition includes both the capacity for comprehension and the mental act or activity of knowing. It comes from the Latin cognoscere, which means “to know or recognize.” As a result, it calls for a variety of mental processes, including reasoning, perception, imagination, and memory [9]. The doctor employs a methodical strategy that determines the presence and severity of the disturbance, the implicated cognitive domains, the potential underlying factors, and the most effective interventions to evaluate and treat a patient who presents with a cognitive complaint. Cognitive issues are a frequent source of problems in clinics because they make it difficult for patients to complete daily tasks, such as working, and they also place a heavy functional and emotional strain on the patients themselves and their family members [10]. It is widely known that CNS viral infections can cause temporary or permanent cognitive impairment [11] through a variety of mechanisms, the most common of which are inflammatory processes and cerebral hypoxia [12, 13].

It is not surprising that as the COVID-19 pandemic spreads, neuropsychiatric symptoms, such as cognitive impairment, worsen [14, 15]. This has been reported by so many studies that found long-term cognitive decline following COVID-19 infection [16–18]. There have been a number of different mechanisms put forth, including direct tissue damage brought on by viral infection and duplication in the nervous system, indirect effects brought on by neural immunopathology brought on by nonspecific immune function stimulated by the viruses, or a combined effect including consequences of the infectious disease [19]. Additionally, it was discovered that SARS-CoV-2 may attach to angiotensin-converting enzyme 2 receptors on cerebral vascular endothelial cells and enter the brain through the olfactory bulb. As a result, the virus may directly cause intrinsic harm to the central nervous system or indirectly through systemic infection elsewhere in the body [20]. Furthermore, ApoE e4 genotype, a known genetic risk factor for dementia and Alzheimer’s disease, was found to be linked to an elevated likelihood of a severe COVID-19 infection which adds to the relation between COVID-19 infection and cognitive decline [8]. COVID-19 and psychiatric diseases were found to be associated in both directions. Infection with COVID-19 has both mental consequences and antecedents. Anxiety, depression, and insomnia are among the new psychiatric illnesses that survivors are more likely to develop. Besides, patients with pre-existing psychiatric problems are at a higher risk of COVID-19 because of their lowered capacity for self-care and their frequent contact with medical personnel, which increases their vulnerability to viral infection [21]. The purpose of this study was to assess the impact of COVID-19 infection on cognitive functions in a sample of recovered Egyptian COVID-19 patients.

## Methods

A longitudinal observational follow-up study was done in Ain Shams University Hospitals where the researcher has attended the chest clinic twice weekly for 1 year. Patients have been followed up for 1, 3, and 6 months following their recovery from COVID-19 infection after taking their written informed consent. The sample size was calculated using PASS program version 15, setting the type-1 error (*α*) at 0.05 and the confidence interval width at 0.1 (margin of error 5%). Result from previous study [22] showed that 2.6% of cases had post-COVID-19 cognitive impairment. Calculation according to these values produced a minimal sample size of 39 cases. However, we included 54 cases to compensate for dropout cases with drop of 4 cases due to incomplete data, so the study sample included 50 patients. They included male and female patients, aged 18–65 years old with confirmed COVID-19 infections.

COVID-19 infection has been confirmed according to case definition of confirmed cases as per WHO criteria as either of the following: (A) a person with a positive polymerase chain reaction (PCR), regardless of clinical criteria or epidemiological criteria and (B) a person meeting clinical criteria AND/OR epidemiological criteria with a positive professional-use or self-test SARS-CoV-2 antigen-RDT {Ag-RDT (= antigen-detection rapid diagnostic test): are available for use by trained professionals or for self-testing by individuals: (A) professional-use SARS-CoV-2 antigen-RDT: it is WHO EUL (Emergency Use Listing)-approved Ag-RDT, in which sample collection, test performance, and result interpretation are done by a trained operator. (B) self-test SARS-CoV-2 antigen RDT: WHO EUL-approved Ag-RDT in which sample collection, test performance, and result interpretation are done by individuals by themselves.}.

Those clinical criteria included acute onset of fever AND cough OR acute onset of any three or more of the following signs or symptoms: fever, cough, general weakness/fatigue, headache, myalgia, sore throat, coryza, dyspnea, and nausea/diarrhea/anorexia, while epidemiological criteria included contact of a probable or confirmed case or linked to a COVID-19 cluster (a group of symptomatic individuals linked by time, geographic location, and common exposures, contacting at least one PCR-confirmed case or at least two epidemiologically linked, symptomatic persons with positive professional use OR self-test Ag-RDT). On the other hand, a probable case of COVID-19 infection is defined as either one of 2 options: (A) a patient who meets clinical criteria AND is a contact of a probable or confirmed case or linked to a COVID-19 cluster and (B) death, not otherwise explained, in an adult with respiratory distress preceding death AND who was a contact of a probable or confirmed case or linked to a COVID-19 cluster.

Patients with previous known cognitive impairment; with any central nervous system diseases; with a known history of mental disorders; with hepatic, renal, or heart failure; and with hearing or visual impairments were excluded from this study. The Faculty of Medicine Ain Shams University Research Ethics Committee (FMASUREC) has approved the study design. A written informed consent has been obtained from all patients.

All cases included in the study have been subjected to a designed questionnaire to collect demographic data such as age, gender, educational level, and occupation as well as clinical presentation of the patients. Also, family history of any cognitive impairment or psychiatric disorders as well as affection of any other family member with COVID-19 infection has been assessed. Furthermore, Wechsler Adult Intelligence Scale (WAIS) was applied to provide an overall assessment of general cognitive functioning and to rule out specific instances such as intellectual giftedness or disability for patients [23]*.* Besides, Wechsler Memory Scale-Revised (WMS-R) was used to assess memory for verbal and figural stimuli, meaningful and abstract material, and delayed as well as immediate recall [24]. Furthermore, the Wisconsin Card Sorting Test (WCST) was applied to diagnose problems associated with the frontal lobe of the brain. It measures the ability to carry out certain types of abstract reasoning, particularly the ability to change problem-solving strategies as needed in addition to being easy to administer [25]. The rationale beyond use of these tests specifically came from the finding by prior literature that the most affected cognitive domains following COVID-19 infection were the executive functions and memory by its different types (immediate, episodic, and working memory) [16, 17, 26]. Additionally, structured clinical interview for DSM-IV, which is simpler and less time-consuming, was used to exclude all major psychiatric disorders as psychosis, bipolar disorders, substance use disorders, and obsessive–compulsive and related disorders (that was mentioned in the exclusion criteria) [27, 28]. Yet, finding that depression and anxiety symptoms is among the commonest psychiatric sequelae of COVID-19 promoted to study the relationship between severity of anxiety and depressive symptoms and cognitive functions in recovered COVID-19 patients through specified tools as Hamilton Anxiety Rating Scale (HAM-A) [29, 30] and Beck’s Depression Inventory to detect subthreshold symptoms [31].

### Statistical analysis

The collected data was analyzed using Statistical Package for Social Science (SPSS 25). Data were presented, and suitable analysis was done according to the type of data obtained for each parameter including the following:

1. Mean, standard deviation (± SD) for continuous data.
2. Parametric tests including Shapiro test were used to test for normality.
3. Frequency and percentage of categorical data.
4. General linear model using a generalized estimating equation.
5. Correlation analysis (using Spearman’s method) which is a nonparametric measure of rank correlation (statistical dependence between the rankings of two variables). The correlation coefficient defines the strength (magnitude) and direction (positive or negative) of the relationship between two variables.

## Results

### Information on demographics

Fifty participants were enrolled in this study who were patients in Ain Shams University Hospitals and were followed up following their recovery from COVID-19 infection. Besides, there were 18 males by a percentage of 36% and 32 females by a percentage of 64%. Additionally, there were 40 participants of high education level by a percentage of 80% and 10 participants with diplomas by a percentage of 20%. Furthermore, there were 41 nonsmoker participants by a percentage of 82% and 9 smoker participants with a percentage of 8%. Additionally, 44 participants had no comorbidities by a percentage of 88%, while 6 participants had comorbidities by a percentage of 12% in the form of diabetes mellitus, hypertension, deep venous thrombosis, and psoriasis as depicted in (Table 1).

**Table 1:** Demographic data of the studied group

		**Mean ± SD**	**Range**
Age		35.4 ± 10.63	23–58
		N	%
Sex	Male	18	36.0%
	Female	32	64.0%
Educational level	Diplomas	10	20.0%
	Higher education	40	80.0%
Smoking	Nonsmoker	41	82.0%
	Smoker	9	18.0%
Comorbidities	No	44	88.0%
	Yes	6	12.0%

*SD* Standard deviation

### Criteria of the studied patients

Among the studied group, 44 participants developed clinical symptoms suggestive of COVID-19 by a percentage of 88%, 18 participants had contact with confirmed COVID cases all over the past 14 days before appearance of the clinical symptoms by a percentage of 36%, 29 participants showed + ve PCR test by a percentage of 58%, and 26 participants showed diagnostic standard investigation (laboratory tests as complete blood picture “CBC,” INR, ESR, CRP, ferritin in addition to CT chest) results by a percentage of 52% as shown in Table 2.

**Table 2:** Selection criteria of the studied group

Selection criteria of the studied group		**Number**	**%**
Clinical symptoms	No	6	12.0%
	Yes	44	88.0%
Contact with confirmed COVID cases	No	32	64.0%
	Yes	18	36.0%
Positive PCR	No	21	42.0%
	Yes	29	58.0%
Standard investigations (laboratory tests as CBC, ESR, ferritin, and CT chest)	No	24	48.0%
	Yes	26	52.0%

### Psychometric scales done for the studied group

Regarding Hamilton Anxiety Scale (HAS), it ranges from 0 to 40 with a mean of 12.4 ± 8.4. Also, Beck Depression Inventory (BDI) ranges from 0 to 36 with a mean of 13.62 ± 9.83, while, Wechsler Adult Intelligence Scale (WAIS), total intelligence quotient (TIQ) ranges from 102 to 125 with a mean of 113.49 ± 9.59 as depicted in Table 3.

**Table 3:** Psychometric scales done for the studied group

Psychometric scale	**Mean ± SD**	**Range**
Hamilton Anxiety Scale	12.4 ± 8.4	0–40
Beck Depression Scale	13.62 ± 9.83	0–36
Wechsler Adult Intelligence Scale (TIQ)	113.49 ± 9.59	102–125

### Wisconsin Card Sorting Test (WCST)

For preservative errors, on comparison between the 1st, 2nd, and 3rd follow-ups, the 2nd follow-up showed nonsignificant comparison to the 1st with a *P*-value of 0.062, while the 3rd showed highly significant comparison to the 1st with a *P*-value of 0.000. While, for non-preservative errors, on comparison between the 1st, 2nd, and 3rd follow-ups, the 2nd follow-up showed significant comparison to the 1st with a *P*-value of 0.001, while the 3rd showed highly significant comparison to the 1st with a *P*-value of 0.013 as shown in Table 4. While for conceptual level responses parameter, it could be noticed that on comparison between the 1st, 2nd, and 3rd follow-ups, both the 2nd and the 3rd follow-ups showed nonsignificant comparison to the 1st with a *P*-value of 0.098 and 0.618, respectively, as represented in Table 5.

**Table 4:** Preservative errors parameter

**Preservative errors**	**Mean ± SD**	**95% CI**	***p*-value**
1st follow-up	15.2 ± 1.9	6.96–10.3	Ref
2nd follow-up	11.85 ± 1.54	8.84–14.86	0.062
3rd follow-up	8.63 ± 0.85	11.47–18.93	0.000
**Non-preservative errors**	**Mean ± SD**	**95% CI**	***p*** **value**
1st follow-up	12.9 ± 1.32	6.35–11.62	Ref
2nd follow-up	9.21 ± 0.99	7.27–11.14	0.001
3rd follow-up	8.99 ± 1.34	10.32–15.48	0.013

*CI* Confidence interval, *P*-value probability value

**Table 5:** Conceptual level responses parameter

Con level response	Mean ± SD	95% CI	*p*-value
1st follow-up	63.94 ± 2.24	63.62–66.82	Ref
2nd follow-up	68.49 ± 1.77	65.02–71.97	0.098
3rd follow-up	65.22 ± 0.82	59.54–68.34	0.618

### Wechsler Memory Scale-Revised (WMS-R) parameters

For digit span parameter, on comparison between the 1st, 2nd, and 3rd follow-up, the 2nd follow-up showed a significant comparison to the 1st with a *P*-value of 0.037, while the 3rd follow-up showed a highly significant comparison with a *P*-value of 0.001. While regarding visual memory parameter, on comparison between the 1st, 2nd, and 3rd follow-ups, both the 2nd and the 3rd follow-ups showed nonsignificant variation to the 1st with a *P*-value of 0.061 and 0.134, respectively. Besides, for verbal paired associates 1 (VPA1) parameter, on comparison between the 1st, 2nd, and 3rd follow-ups, both the 2nd and the 3rd follow-ups showed a highly significant comparison to the 1st follow-up (*P*-value of < 0.001) for both as shown in Table 6.

**Table 6:** Wechsler Memory Scale-Revised (WMS-R) parameters

**Digit Span**	**Mean ± SD**	**95% CI**	***p*-value**
1st follow-up	12.69 ± 0.49	13.3–15.41	Ref
2nd follow-up	13.45 ± 0.39	12.69–14.21	0.037
3rd follow-up	14.35 ± 0.54	11.74–13.65	0.001
**Visual memory**	**Mean ± SD**	**95% CI**	***p*-value**
1st follow-up	14.67 ± 0.45	14.39–16.47	Ref
2nd follow-up	15.5 ± 0.43	14.66–16.34	0.061
3rd follow-up	15.43 ± 0.53	13.78–15.56	0.134
**Verbal Paired Associates 1**	**Mean ± SD**	**95% CI**	***p*-value**
1st follow-up	14.67 ± 0.63	16.39–19.16	Ref
2nd follow-up	16.95 ± 0.58	15.81–18.08	0.001
3rd follow-up	17.77 ± 0.71	13.44–15.9	0.001

### Correlations between anxiety symptoms, depressive symptoms, and Wisconsin Card Sorting Test (WCST) parameters

There was no significant correlation between Hamilton Anxiety Scale (HAS) and any parameter of Wisconsin Card Sorting Test (WCST). In addition, there was no significant correlation between Beck Depression Inventory (BDI) and any parameter of Wisconsin Card Sorting Test (WCST) as shown in Table 7.

**Table 7:** Correlations between anxiety, depression, and Wisconsin Card Sorting Test (WCST) parameters

	**Hamilton Anxiety Scale**		**Beck Depression Inventory**	
	**Spearman’s Rho**	***p*-value**	**Spearman’s Rho**	***p*-value**
Total correct	− 0.038	0.796	− 0.072	0.624
Total errors	0.088	0.549	0.015	0.920
Preservative responses	0.123	0.400	0.057	0.698
Preservative errors	0.114	0.436	0.052	0.724
Non-preservative errors	0.025	0.867	− 0.027	0.854
Conceptual level response	0.034	0.819	− 0.137	0.349
Categories completed	− 0.136	0.352	− 0.160	0.271
Trials to complete 1st category	0.088	0.549	0.157	0.283
Failure to maintain set	− 0.003	0.984	− 0.085	0.561

### Correlations between anxiety symptoms, depressive symptoms, and Wechsler Memory Scale-Revised (WMS-R) parameters

There was no significant correlation between Hamilton Anxiety Scale (HAS) and any parameter of Wechsler Memory Scale-Revised (WMS-R). As well, there was no significant correlation between Beck Depression Inventory (BDI) and any parameter of Wechsler Memory Scale-Revised (WMS-R) as shown in Table 8.

**Table 8:** Correlations between anxiety, depression, and Wechsler Memory Scale-Revised (WMS-R) parameters

	**Hamilton Anxiety Scale**		**Beck Depression Inventory**	
	**Spearman’s Rho**	***p*-value**	**Spearman’s Rho**	***p*-value**
Digit span 1	− 0.025	0.864	0.057	0.697
Visual memory 1	− 0.217	0.134	− 0.103	0.481
1st verbal paired associates 1	− 0.139	0.341	− 0.051	0.730
1st verbal paired associates 2	− 0.249	0.085	− 0.069	0.635
1st visual paired associates 1	− 0.091	0.533	− 0.032	0.829
1st visual paired associates 2	− 0.035	0.809	0.078	0.596

## Discussion

A combination of signs known as post-COVID syndrome may last longer than 3 weeks after the start of an acute COVID infection. These symptoms may persist for up to 6 months or more after SARS-CoV-2 infection has cleared up [32]. This study was conducted to evaluate the COVID-19 virus infection’s post-remission cognitive consequences in people who survived.

Upon analyzing demographics, our results revealed that 64% of the patients were female, non-smoking with higher education. According to reports, a person’s physical, cognitive, economic, emotional, mental, function, and spiritual state all play a role in their health-related quality of life [33]. Contrarily, a small number of research, carried out prior to the COVID-19 outbreak, found no association between sex and patients’ quality of life in terms of their health [34, 35]. This could be attributed to different psychometric tools used and differences in sample size of those studies. Nevertheless, women’s lower levels of physical activity, particularly in underdeveloped nations [36], would be linked to their lower quality of life [37].

In the present study, various psychometric scales were conducted in the tested sample of Egyptian patients including Hamilton Anxiety Scale, Beck Depression Scale, and Wechsler Adult Intelligence Scale which showed 12.4 ± 8.4, 13.62 ± 9.83, and 113.49 ± 9.59, respectively. Other group of researchers applied the Arabic version of fear of COVID-19 scale with a good reliability [38]. Furthermore, results from a psychometric analysis of the COVID-19 anxiety and fear scales among Italians reveal that variations in gender, age, marital status, and educational attainment have an impact on the scales’ scores. Moreover, it appears that exposure to COVID-19 and its subsequent deaths have a profoundly negative impact on people’s mood, potential risk, and depressive symptoms [39]. Other groups tried to test and validate other scales, e.g., COVID-19 Impact Scale (CIS), Preventive COVID-19 Infection Behaviors Scale (PCIBS), and Depression Anxiety Stress Scale-21 (DASS-21) to assess impact of COVID-19 with a notable success and many limitations [40, 41]. These variations could be explained through different age range and psychometric tools used to assess anxiety and fear symptoms that are different from those used in the present study, including usage of structured clinical interview for DSM-IV to exclude depression and anxiety disorders.

In the present study, various parameters of Wisconsin Card Sorting Test were tested including for preservative errors, non-preservative errors, and conceptual level responses along 1st, 2nd, and 3rd follow-ups of COVID-19 infection revealing variations between different follow-ups. Shields et al. [39] evaluated executive functioning in anxious people using the Wisconsin Card Sorting Test. Men are much more impacted than women, according to their research, which shows that there are gender-specific impacts on cognitive control in humans that comes in contrast to the present study. This can be explained by their larger sample size and the finding that females are the majority of the present study’s sample. Moreover, results of the study by Lucas et al. [40] who evaluated functional and microstructural brain abnormalities, fatigue, and cognitive dysfunction after COVID-19 infection came in line with our results and found cognitive impairment, especially memory problems in these individuals. It is well recognized that mental health issues, such as anxiety, can impair cognitive function [41]. In line with the results of the present study, Hetong and his colleagues who studied the landscape of cognitive functions in recovered COVID-19 patients found a potential cognitive dysfunction in patients with COVID-19 that are linked to the inflammatory process, specifically sustained attention [42]. This could be attributed that Hetong used not only digit span test that is done in the current study but also other psychometric tools as trail-making test as well as laboratory testing of interleukin levels. However, research study found a range of statistical variations across groups throughout a variety of follow-ups; thus, the results need to be recoded and applied carefully.

In the current report, different parameters of Wechsler Memory Scale-Revised (WMS-R) were applied including digit span, visual memory, and verbal paired associate parameters along 1st, 2nd, and 3rd follow-ups of COVID-19 infection, where both the 2nd and the 3rd follow-ups showed a highly significant comparison to the 1st follow-up in digit span and verbal paired associate parameters. Meanwhile, both the 2nd and the 3rd follow-ups showed nonsignificant difference to the 1st in visual memory parameter. Almeria et al. [10] applied Wechsler Memory Scale as one of the used scales to examine neurocognitive difference in patients in Spain to illustrate that cognitive complaints were accompanied with anxiety and depression. The differences from results of the present study can be explained through being of different races that consequently lead to different timing of COVID-19 infection with different underlying COVID-19 strains as well as the possible role of genetic variations.

The present results revealed nonsignificant correlations between anxiety symptoms, depressive symptoms, and Wisconsin Card Sorting Test as well as Wechsler Memory Scale-Revised parameters. However, Ollila et al. [43] reported that cognitive functioning was significantly affected in long-term ICU patients in Finland. It is worth mentioning that this study was conducted exclusively on hospitalized patients who were assessed only once, while the majority of the present study’s patients were nonhospitalized in addition to being assessed and followed up for 3 successive times. Furthermore, cognitive impairment was present in 22% of participants in a recent meta-analysis of 43 trials that included individuals evaluated 12 or more weeks following COVID-19 infection [44]. In a review concentrating on quantitative neuropsychological testing on COVID-19 participants, 15–80% of people had global cognitive problems. On average, concentration and executive function issues were found in seven out of twelve investigations. Memory deterioration was seen in three of the four investigations [45]. This could be correlated to the different timings of assessment of the patient that reflects their clinical phase of COVID-19 infection, being in acute or subacute stages.

## Conclusions

The current research demonstrated that there is negative impact of COVID-19 infection on cognitive functions in Egyptian recovered COVID-19 patients which improves gradually by time as detected after 3 and 6 months by following up the patients using the Wisconsin Card Sorting Test and Wechsler Memory Scale-Revised parameters.

## Data Availability

The manuscript contains all of the data that were created or examined during this study.
